# Making Every Child a Chemist

**Published:** 1889-10

**Authors:** 


Making Every Child a Chemist.—In the chemical section of the British Association one of the most animated discussions this year arose on the report of the committee appointed to inquire into and report upon the present methods of teaching chemistry. The committee was appointed at the Manchester meeting, and submitted a preliminary report at Bath; but it is since then that their ideas have taken definite shape, and the working scheme which they submitted at Newcastle-on-Tyne is mainly the product of Professor H. E. Armstrong's brain. Little notice has been given to this report, and apparently for this reason a leaded article, calling attention to it, appears in the last number of *Nature* over the initials W. R. D., in which there is little difficulty in recognizing Professor W. R. Dunstan, the secretary of the committee. This article is written in a forcible style, and is especially addressed to teachers of science, so that it is likely to earn for the report the attention which it deserves. It will be remembered that last year's report referred to the manner in which chemistry is taught in 500 higher-grade schools. To complete the record Professor Smithells has prepared for the committee similar statistics regarding the position occupied by chemistry in public elementary schools which are controlled by the State education departments. He shows that as a rule chemistry is not taught on the proper lines, and the subject has failed to provide that mental training which it should be the main object of elementary teaching to develop. Here is the kernel of the whole matter, and it will favour brevity to keep these facts in view. Professor Dunstan is of opinion that "the mental discipline afforded by chemical investigation, in developing the powers of observation and inference, and in teaching the correct use of hypothesis, is not less conspicuous than the many applications of which the science is susceptible, both in industrial and 'common' life." Hitherto the chemical teaching of the schools has signally failed in exercising any mental discipline, and why? There are several causes of failure. The first is that the scholastic curriculum is already overloaded to a serious degree. Children of twelve, of ordinary parts, have at least eight subjects to study, viz., English grammar, English history, geography, arithmetic, reading aloud, writing and dictation, drawing, and Latin, if they have progressed fairly. These are taught in about twenty five hours per week, five hours per day being the utmost that young heads can afford for mental concentration. It follows, therefore, that the study of science must be in the nature of mental recreation rather than of mental discipline, and so hitherto has it been treated in practice. A few desultory lessons from a junior master who is enamoured of chemistry, or one or two hours per week from an itinerant science master, have been all that could be squeezed into the curriculum; the lessons have consequently been more of an entertaining than an instructive character. The net result generally has been spasmodic demands for pennyworths of sodium, red precipitate, and so forth. In view of this experience it is somewhat surprising that the British Association committee should have come to the conclusion that a change in the method of teaching science would give it a better footing in the scholastic curriculum. Professor Dunstan correctly says that in order to make science teaching successful "a fair share of the school time must be devoted to the subject, and a larger number of teachers must be employed than is usually the case." In other words, room must be made for it in the curriculum—a subject must be sacrificed—and the State will have to increase the education grant by fully half a million a year. In return for that a course of instruction in science will be given somewhat similar to Professor Armstrong's which Professor Dunstan thus outlines:—

The course is divided into six "stages." Stage I. deals with lessons on common and familiar objects; the classification of these according to their uses and origin; elementary physiography and *Naturkunde* (geography and natural history.) Stage II., with lessons in measurement. Stage III., with studies of heat on things in general; of their behaviour when burnt. Stage IV., the problem stage; to determine what happens when iron rusts; to determine the nature of the changes which take place when substances are burnt in air; to separate the active from the inactive constituent of air; to determine the composition of chalk; to determine the composition of washing soda; and much other similar work. Stage V., the quantitative stage: study of the quantitative composition of some of the substances which have already been qualitatively examined. Stage VI., studies of the physical properties of gases in comparison with those of liquids and solids; the molecular and atomic theories, and their application.

The committee honestly submit their opinion that "in order that the plan shall produce its full educational effect, the instruction should be commenced at an early age, and be extended to every child in the school. They do not desire to bring forward physical science as a substitute for any of the other principal subjects of study, but they ask that, like these subjects, it should be looked upon everywhere as a necessary part of education, and that it should receive a due share of the time devoted to school work" Let it be admitted that science teaching is a splendid tool for promoting mental discipline; what, then, is there to sacrifice for it? It would be a splendid thing for science teachers if every child in the realm had to come under their guidance. We should have as many of them as of head masters and a new tax would have to be created to pay them, not to mention the new corps of science-inspectors which, the committee hints, may be formed from "the professors and lecturers in university colleges and other educational institutions" This is a part of the subject which the British Association committee have not considered. We wish they would.

